# Medicines dispensing practice during the era of COVID-19 pandemic: a commentary

**DOI:** 10.1186/s40545-020-00285-5

**Published:** 2021-01-04

**Authors:** Gemmechu Hasen, Rashed Edris, Gadisa Chala, Yesuneh Tefera, Hawi Hussen, Tamirat Tekassa, Sultan Suleman

**Affiliations:** 1 School of Pharmacy, Faculty of Health Sciences, Jimma University, P.O. Box: 378, Jimma, Ethiopia; 2 Laboratory of Drug Quality (JuLaDQ), Jimma University, Jimma University, Jimma, Ethiopia; 3 Department of Environmental Health, Faculty of Health Sciences, Jimma University, Jimma, Ethiopia

**Keywords:** Dispensing practice, COVID-19, Retail outlet, Jimma Town

## Abstract

The coronavirus disease 19 (COVID-19) pandemic is putting a huge strain on healthcare systems and is a turning point for the beginning of a global health crisis of an unprecedented condition. As such, the provision of quality pharmacy services particularly, dispensing practice with pre-existing challenges in resource-limited settings is a grave concern in the era of the COVID-19 pandemic. Thus, in this commentary we described the pattern of dispensing practice in the midst of the COVID-19 pandemic by evaluating the current condition of drug dispensing practice in drug retail outlets of Jimma Town.

## Background

Coronavirus disease 2019 (COVID-19) is a global pandemic caused by the severe acute respiratory syndrome coronavirus-2 (SARS-CoV-2) that was first identified in the city of Wuhan, Hubei Province, China, at the end of 2019 [1]. On January 30, 2020 the World Health Organization (WHO) declared the outbreak as a Public Health Emergency of International Concern, and has reached a new dimension with a higher death ratio [2]. Since then, the virus is putting a huge strain on healthcare systems and is a turning point for the beginning of a global health crisis of an unprecedented nature and scale [3]. In response to this, WHO recommended practical actions to reorganize and safely maintain access to high-quality, essential health services in the context of the COVID-19 pandemic [4]. Accordingly, the provision of medications, management of emergency health conditions and common acute presentations that require time-sensitive intervention are among the high-priority categories of essential health services, which alert pharmacist’s role as the providers of an essential health service across the globe [5]. The pharmacists have always been the most accessible health care provider; this is especially true in the era of COVID-19 as they remain on the frontline of public health by serving as direct points of access for their patients [6, 7]. In many countries, at a national level, pharmacies are defined as essential services that remain open 7 days a week and accessible to the public to manage the COVID-19 pandemic even during lockdown restrictions [8]. Moreover, as the first point of contact for their easy access and availability, pharmacists have been involved in early detection, referral, and facilitation of implementing various government measures aimed at preventing and/or slowing down the spread of the pandemic [9]. On other hand, pharmacists, like any other health professionals, are responsible for providing safe and ethical care to the public at all times in accordance with professional standards [10]. Thus, besides the aforementioned responsibilities, pharmacists are mandated to promote rational use of medicines through appropriate drug dispensing practice, and offer emotional support while identifying those in need of further care during this pandemic [11]. However, providing quality pharmacy services particularly, dispensing practice with pre-existing challenges is a grave of concern [12], which obviously aggravated by the spread of the COVID-19 pandemic. Therefore, this commentary is aimed to describe the pattern of dispensing practice in the midst of the COVID-19 pandemic by evaluating the current condition of drug dispensing practice in drug retail outlets of Jimma Town. The two study approaches encompassing; face-to-face interviews and simulated patients visits were employed, the full methodology is presented as Additional file 1.

## Discussion

Everyone agrees that the COVID-19 pandemic is putting an unprecedented impact on health systems, especially pharmacy services as pharmacists are the first point of contact with patients and/or caregivers that could seriously affect quality of medication dispensing practice. Thus, the present study reported the current condition of dispensing practice in drug retail outlets of Jimma Town during era of the COVID-19 pandemic. The total score obtained by the dispensers was calculated from the Likert scale structured questions, and the current status of the dispensing practice was established for the dispensers, and full results are presented as Additional file 2.

Accordingly, the results obtained from dispenser’s response were compared with the simulated patients, and crucial discrepancies were found. As stated by dispensers the average dispensing time was 4.7 ± 2.6 min, and the dispensing time measured by simulated patients using stop watch was 1.71 ± 0.632 min, which are consistent with WHO recommendation that pharmacists should spend at least 3 min in orienting each patient [13]. Moreover, the dispensing time is very low when compared with results reported in various regions of Ethiopia prior to the COVID-19 pandemic [14–16]. This shorter dispensing time in current report is not amazing, as small number of dispensers provided inadequate medication counselling like, possible contraindications, purpose of the prescribed drug, and importance of dispensed drug compliance, and none of them told the storage condition of prescribed drug. This situation could leads to wrong use of medication and irrational use of drugs, thus burdening the individuals and health care system [17]. Moreover, the inadequate medication counselling may also contribute to wastage of drugs dispensed due to poor communication about their usage [18]. In a view of COVID-19, the shorter time that implicates inadequate medication counselling may be associated with dispenser’s fear of contracting the virus.

On other hand, the simulated patients revealed that inadequate labelling of crucial information was less and/or missed by majority of dispensers. This finding reveals the impacts of COVID-19 pandemic on dispensing practice. Since, the label on dispensed drugs is very crucial to give patients clear and concise information about the use of the medication, the failure to appropriate labelling could increase chance of drug toxicity, and therapeutic failure [14].

In our report, the experience of the dispensers and time spent in drug outlet were significantly associated with the status of dispensing practice. This could be related to commitment of experienced dispensers as they may spend more time in the outlets during business hours to satisfy his or her customers. Moreover, workload (number of patients per day) and dispensing time for a single drug were associated with the condition of the dispensing practice in the midst of the COVID-19 pandemic. These may be related to a reduced time dispensers spend with each customer as they may work in a more commercial manner or the fear of the pandemic. This may also be related to the extension of pharmacists role to fight against the pandemic.

## Conclusions

In conclusion, the COVID-19 pandemic is highly affecting the pattern of dispensing practice as revealed by unsatisfactory dispensing practice in drug retail outlets of Jimma Town. Moreover, the experience of dispenser, time spent in drug outlet by dispenser, workload, satisfaction of dispenser, and dispensing time for a single drug were significantly associated with current conditions of dispensing practice. Therefore, the regulatory authority should urgently develop strategy for drug dispensing practice in the context of the COVID-19 pandemic to promote the rational use of medicines.

## Supplementary Information

**Additional file 1:** Methodology.

**Additional file 2:** Results.

## Data Availability

The supporting documents for this study can be made available from the corresponding author upon request.
