# Supplementary material for: Medicines dispensing practice during the era of COVID-19 pandemic: a commentary
Source: J Pharm Policy Pract. 2021 Jan 4;14:1. doi: 10.1186/s40545-020-00285-5 (PMC7780590; doi:10.1186/s40545-020-00285-5)
Supplement: Supplementary file 1 — Additional file 1: Methodology. [file 40545_2020_285_MOESM1_ESM.docx]

**Methodology**

**Study design and setting**

All practicing dispensers from Jimma town private drug outlets (n=57) were invited to participate. Out of 57 [Pharmacy (n=21), Drug store (n=33)] drug retail outlets, dispensers from 48 drug outlets were participated during study period between September and October, 2020. Jimma town is located 346 km south west direction of the capital city Addis Ababa, Ethiopia. Jimma University Research & grants office approved the research. We received a letter of permission from Jimma Regional Health bureau. To collect data we obtained written informed consent of the study participants prior to interviews. The right of participants to withdraw from an interview at any time was maintained. We included all dispensers from private drug outlet who have at least 2 month experience have given full consent, and available during the study period. We translated an English version of the questionnaire to local languages, Afaan Oromo and Amharic, and back translated to English. The data collection tools were also pretested for both study approaches. The approaches of the study encompass face to face interviews with dispensers and simulated patients to dispensing practice. To prevent the spread of COVID 19, the WHO recommendations, wearing of mask, physical distance at 2 meter, hand sanitizers were applied by trained data collectors during data collection. For face to face interview, the questionnaire was adopted from FMHACA (Food, Medicine and Healthcare Administration and Control Authority of Ethiopia) standard guideline for medication dispensing practice (18), and previous study conducted on the dispensing practice (19). Accordingly, fifteen open ended and six structured questions were developed for the interview. The structured statements (Likert items) were: strongly agree, agree, neutral, disagree and strongly disagree. Moreover, simulated patients were used to compare the information from the interview with the actual practice. Three Master of Science students from School of Pharmacy, Jimma University were role played. A standard prescription was used. Information about the prescribed medication counselling and drug labelling were filled immediately after leaving the drug retail outlets, and dispensing time was recorded using stop watch. Simulated patients data collection was after two weeks of face to face interview. Scenario, 30 years old male patient enters to the drug retail outlet with a prescription of Ciprofloxacin 500 mg tablet, 1 tab, twice daily for typhoid treatment that written for 28 years old female patient. Before leaving the outlets, he asked the dispenser for an antacid tablet for her upset stomach. He also asked the dispenser to give omeprazole for himself.

**Statistical analysis**

Each questionnaire was checked for completeness and accuracy before entering the data into SPSS. The data was cleaned for errors and missing data, coded, categorized and sorted to facilitate the analysis and entered into the computer. We analysed the data using SPSS Version 21.0 (Chicago, SPSS Inc.). The results were summarized using in text, percentages, tables and bar graphs. Each Likert item was rated on a 1-5 response scale; where strongly agree=5, agree-4, neutral=3, disagree=2, strongly disagree=1. Accordingly, the total score for each dispenser were calculated out of 30 score (6 questions with maximum of 5 score), and presented as percentages. The score was graded to acceptable (≥ 50% score) and unacceptable (<50%) score of dispensing practice (20). Chi square was used to determine association between level of current dispensing practice and variables of perceptions and socio-demographic characteristics of dispensers at 5% (p< 0.05) level of significance.

Operational definitions and definition of terms

**Dispensing practice:** refers to a process that correct medicine is delivered to the right patient, in the required dosage and quantities, with clear instructions, and in package that maintains an acceptable potency and quality of the medicine (1).

**Drug retail outlets:** refers to any establishment where registered or over-the counter drugs are dispensed directly to the general public on a retail basis (2).

**Simulated patients:** is a technique of assessing dispensers, dispensing practices by visiting the drug retail outlets after training specific patient scenario (3).

**Acceptable dispensing practice:** earning ≥ 50% score for current condition of dispensing practice Likert scale questions.

**Unacceptable dispensing practice:** earning <50% score for current condition of dispensing practice Likert scale questions

**References**

1. EFMHACA. Manual for Medicines Good Dispensing Practice. 2012 USAID.
2. Riley, Pamela, Sean Callahan, and Mike Dalious. Regulation of Drug Shops and Pharmacies Relevant to Family Planning: A Scan of 32 Developing Countries. Bethesda, MD: Sustaining Health Outcomes through the Private Sector Plus Project, Abt Associates Inc. July 2017.
3. Netere AK, Erku DA, Sendekie AK, Gebreyohannes EA, Muluneh NY, Belachew SA. Assessment of community pharmacy professionals’ knowledge and counselling skills achievement towards headache management: A cross-sectional and simulated-client based mixed study 11 Medical and Health Sciences 1117 Public Health and Health Services. J Headache Pain. 2018; 19(1).
