# Supplementary material for: Medicines dispensing practice during the era of COVID-19 pandemic: a commentary
Source: J Pharm Policy Pract. 2021 Jan 4;14:1. doi: 10.1186/s40545-020-00285-5 (PMC7780590; doi:10.1186/s40545-020-00285-5)
Supplement: Supplementary file 2 — Additional file 2: Results. [file 40545_2020_285_MOESM2_ESM.docx]

**Socio-demographic characteristics of dispensers**

Out of 57 drug retail outlets, 48 drug outlets were participated during study period with 84% response rate. As shown in Table 1, more than half (64.6%) of dispensers were males. The average age of all dispensers was 34.31 ± 8.5 standard deviation, and ranged in age from 20 to 58 years. Regarding year of experience, 35.4% of dispensers reside within less than 5 years of experience category. Majority of (54.2%) dispensers spent less than 8 hours in drug retail outlet. More than half, 56.3%, dispensers interviewed were from drug store. Around 46% of dispensers provide service for 51-100 patients per day (Table 1).

Table 1: Socio-demographic characteristics of dispensers of drug retail outlets of Jimma Town

| **Variables** | | **Frequency** | **Percentage (%)** |
| --- | --- | --- | --- |
| Gender | Female | 17 | 35.4 |
|  | Male | 31 | 64.6 |
| Age | under 25 | 5 | 10.4 |
|  | 26-35 | 27 | 56.3 |
|  | 36-45 | 10 | 20.8 |
|  | above 46 | 6 | 12.5 |
| Educational qualification | Pharmacy | 20 | 41.7 |
|  | Druggist | 28 | 58.3 |
| Year of experience | ≤ 5 years | 17 | 35.4 |
|  | 6-10 years | 15 | 31.3 |
|  | 11-15 years | 9 | 18.8 |
|  | 16-20 years | 4 | 8.3 |
|  | ≥ 21 years | 3 | 6.3 |
| Time spent in drug outlet | < 8 hours | 26 | 54.2 |
|  | ≥ 8 years | 22 | 45.8 |
| Workload (number of pts per day) | ≤ 50 patients | 20 | 41.7 |
|  | 51-100 patients | 22 | 45.8 |
|  | ≥ 101 patients | 6 | 12.5 |
| Type of drug outlets | Pharmacy | 21 | 43.8 |
|  | Drug store | 27 | 56.3 |

**Dispensing practice and perceptions of dispensers**

About 22.9% of dispensers were unsatisfied, and 14.6% were neutral with the way of dispensing practice in the era of COVID 19. From dispenser’s response, the average dispensing spent for prescription of a single drug was 4.7 ± 2.6 minutes (Figure 1). Around 39.6% dispensers think dispensing environment was not safe to practice good dispensing during COVID-19 pandemic. A majority (83.3%) of respondents said it is important to develop a new guideline for dispensing practice in this outbreak (Table 2). Moreover, the majority of respondents applied World Health Organization recommendations such as hand washing (70.8%) and wearing mask (87.5%) to prevent the spread of COVID 19 (Figure 2).

**Table 2: Dispensing practice and perceptions of dispensers during era of COVID 19 Pandemic**

| **Dispensing practice and perceptions of dispensers during era of COVID 19 Pandemic** | | **Frequency** | **Percent** |
| --- | --- | --- | --- |
| Do you satisfied with the way of dispensing practice | Yes | 30 | 62.5 |
|  | No  Unsure | 11  7 | 22.9  14.6 |
| How long do you spend approximately for a prescription of a single drug | ≥ 5 minutes | 27 | 56.3 |
|  | 6-10 minutes | 18 | 37.5 |
|  | ≤ 11 minutes | 3 | 6.3 |
| Do you have training on dispensing practice during COVID pandemic? | Yes | 18 | 37.5 |
|  | No | 30 | 62.5 |
| Do you think dispensing environment safe to practice good dispensing during COVID-19? | Yes | 29 | 60.4 |
|  | No | 19 | 39.6 |
| Is there new dispensing guideline provided from regulatory authority? | Yes | 19 | 39.6 |
|  | No | 29 | 60.4 |
| Do you follow current guideline to prevent spread of COVID 19 pandemic at your retail? | Yes | 44 | 91.7 |
|  | No | 4 | 8.3 |
| Do you think the need of starting new dispensing practice guideline in response to OVICD-19 important? | Yes | 40 | 83.3 |
|  | No | 8 | 16.7 |

**
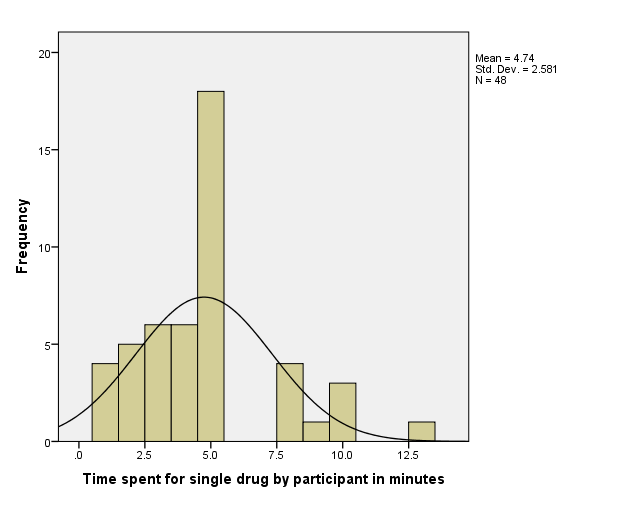
**

Figure 1: Approximate dispensing time obtained from dispensers interview in minutes

Figure 2: Prevention methods for spread of COVID 19 pandemic practiced at retail outlets

**Dispenser’s evaluation of current dispensing practice**

Among study participants, majority of dispensers (77.1%) earned greater than or equal to 50% score for Likert scale questions thus providing acceptable dispensing practice during COVID 19 pandemic (Figure 2). Moreover, the results of the dispensing practice assessment questions of all Likert scale questions were presented on Table 3.

Figure 2: Status of dispensing practice during COVID 19 pandemic at retail outlets

Table 3: Dispenser’s evaluation of current dispensing practice using Likert scale questions

| Level of dispensing practice assessment questions (N=48) | Strongly Agree (%) | Agree (%) | Neutral (%) | Disagree (%) | Strongly Disagree (%) |
| --- | --- | --- | --- | --- | --- |
| Check items like instructions, dose, dosage form, duration, patient, diagnosis, date, age and patients name in each prescription during this pandemic | 70.8 | 25.8 | 4.2 | - | - |
| Provide adequate information for each chronic patient and document their medication profile for each refill visit during this pandemic | 41.6 | 29.2 | 29.2 | - | - |
| Maintain the privacy of each patient/caregiver during dispensing in counselling room during this pandemic while other waiting patients away from dispensing window or Balcony | 25.0 | 43.8 | 8.3 | 18.8 | 4.2 |
| Provide drug information like instruction how to take drugs, dosing of drugs, importance of compliance, possible side effects, drug interaction and storage condition of drugs for each patient/caregiver in separate drug information room or area as a usual during this pandemic | 37.5 | 45.8 | 8.3 | 8.3 | - |
| Record prescriptions details like patient detail, facility detail, diagnosis, medication detail and professional details on log book or electronic during this pandemic | 29.2 | 18.8 | 16.7 | 22.9 | 12.5 |
| During this pandemic, I provide drug with adequate labelling of necessary informations thus patients can understand the information I provide them, I confirm and clarify the understanding of the patient | 52.1 | 27.1 | 14.6 | 6.3 | - |

**Factors affecting current level of dispensing practice**

Statistical analysis of the cross-tabulation between current status of dispensing practice, and variables of perceptions and socio-demographic characteristics of dispensers at 5% (p< 0.05) level of significance revealed that year of experience of dispenser (*χ*2=10.845; *p*-value=0.028), time spent in drug outlet by dispenser (*χ*2=4.157; *p*-value=0.041), workload (number of patients per day) (*χ*2=7.490; *p*-value=0.024), satisfaction of dispenser during pandemic (*χ*2=6.420; *p*-value=0.042), and dispensing time for a single drug (*χ*2=7.011; p-value=0.030) were significantly associated with status of dispensing practice during COVID 19 pandemic (Table 4).

Table 4: Cross-tabulation between current status of dispensing practice and variables of perceptions and socio-demographic characteristics of dispensers

| Variables | | Frequency | Dispensing practice | | *χ*2 | *p*-value |
| --- | --- | --- | --- | --- | --- | --- |
|  |  |  | Acceptable | Unacceptable |  |  |
| Educational qualification | Pharmacy | 20 | 16 | 4 | 1.007 | 0.68 |
|  | Druggist | 28 | 21 | 7 |  |  |
| Year of experience | ≤ 5 years | 17 | 16 | 1 | 10.845 | **0.028^*^** |
|  | 6-10 years | 15 | 13 | 2 |  |  |
|  | 11-15 years | 9 | 5 | 4 |  |  |
|  | 16-20 years | 4 | 2 | 2 |  |  |
|  | ≥ 21 years | 3 | 1 | 2 |  |  |
| Time spent in drug outlet | < 8 hours | 26 | 23 | 3 | 4.157 | **0.041^*^** |
|  | ≥ 8 hours | 22 | 14 | 8 |  |  |
| Workload (number of pts per day) | ≤ 50 patients | 20 | 17 | 3 | 7.490 | **0.024^*^** |
|  | 51-100 patients | 22 | 18 | 4 |  |  |
|  | ≥ 101 patients | 6 | 2 | 4 |  |  |
| Environment safe for dispensing practice | Yes | 29 | 24 | 5 | 1.336 | 0.248 |
|  | No | 19 | 13 | 6 |  |  |
| Satisfaction about the way the practice | Yes | 30 | 26 | 4 | 6.420 | **0.042^*^** |
|  | No | 11 | 8 | 3 |  |  |
|  | Neutral | 7 | 3 | 4 |  |  |
| Training of dispensing during COVID 19 | Yes | 18 | 12 | 6 | 1.769 | 0.184 |
|  | No | 30 | 25 | 5 |  |  |
| Dispensing time | ≥ 5 minutes | 27 | 17 | 10 | 7.011 | **0.030^*^** |
|  | 6-10 minutes | 18 | 17 | 1 |  |  |
|  | ≤ 11 minutes | 3 | 3 | 0 |  |  |

**^*^Statistically significant**

**The results of the simulated patients**

Of the 48 drug retail outlets visited, the simulated patients contacted 93.5% of the outlets, while others were closed during business hours. The mean and standard deviation of the dispensing time for a single drug measured by simulated patients using stop watch were 1.71 ± 0.632 minutes respectively (Figure 3). However, from dispenser’s response, the average dispensing spent for prescription of a single drug was 4.7 ± 2.6 minutes (Figure 1). Around 93% of dispenser refused to provide antacid upon the verbal request of the simulated patients. Moreover, only 4.4% of dispensers agreed to give omeprazole without written prescription. The actual patterns of dispenser counselling and labelling practice were assessed based critical information provided to simulated patients as shown on Table 5 and 6 respectively. Although, 83.3% [(37.5%, strongly agree), (45.8%, agree)] dispensers declared that they provide drug information for each patients (Table 3), the simulated results revealed presence of the discrepancies among the study approaches (Table 5 and 6). Accordingly, only 42.2% of dispensers informed the simulated patients how to take prescribed drug correctly. Around, 26%, 49%, and 6.7% of dispensers advised about possible contraindications, purpose of the prescribed drug, and importance of dispensed drug compliance respectively while none of them told the storage condition of prescribed drug (Table 5). Moreover, 79.2% [52.1% (strongly agree), 27.1% (agree)] dispensers agreed that they provide drugs with adequate labelling of necessary informations. However, the results of simulated patients revealed that only 20% of the dispensers write dosage (strength, dose and total quantity) of the drug on the label. In addition, 44.4% and 42.2% of dispensers labelled the frequency of administration and duration therapy of the dispensed drug respectively, while none of the dispensers wrote date of dispensing storage condition on the label (Table 6).


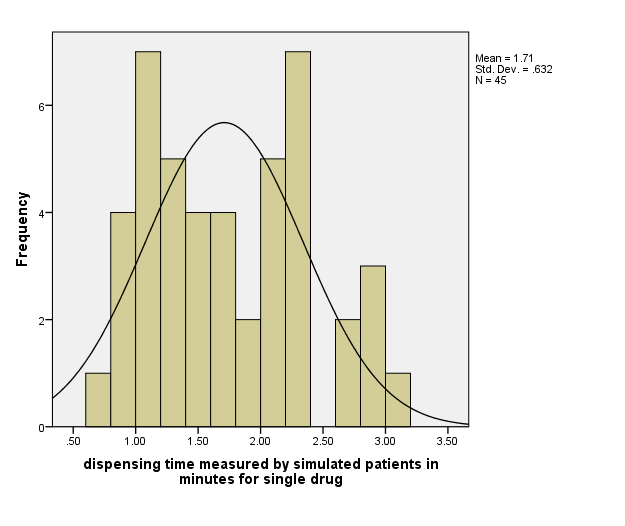
Figure 3: The dispensing time measured by simulated patients with stopwatch

Table 5: Evaluation of information given by the pharmacist to the SP about the prescribed medication counselling

| Type of information given by the dispenser to the SP about the counselling of the prescribed medication (N=45) | Correctly informed (%) | Misinformed (%) | Uninformed (%) |
| --- | --- | --- | --- |
| Does the dispenser told name of the drug to you? | 4.4 | - | 95.6 |
| Does the dispenser advice you how to take the drug? | 42.2 | 24.4 | 33.3 |
| Does the dispenser advice you about the frequency of administration? | 100 | - | - |
| Does the dispenser advice you the purpose of the drug prescribed? | 48.9 | - | 51.1 |
| Does the dispenser advice you the duration of medication has been taken? | 88.9 | 6.7 | 4.4 |
| Does the dispenser advice you about possible food and drug interactions? | 20 | 20 | 60 |
| Does the dispenser advice you about possible side effects? | 100 | - | - |
| Does the dispenser advice you about possible contraindications? (e.g pregnancy) | 26.7 | - | 73.3 |
| Does the dispenser advice you about storage conditions? | - | 2.2 | 98.2 |
| Does the dispenser advice you about importance of dispensed drug compliance? | 6.7 | 2.2 | 91.1 |

Table: 6 Assessment of information written on the label by the dispenser for the simulated patients

| Type of information written on the label by the dispenser for the Simulated Patients (N=45) | Correctly informed (%) | Misinformed (%) | Uninformed (%) |
| --- | --- | --- | --- |
| Does the dispenser write patient name on the label? | - | - | 100 |
| Does the dispenser write dosage (strength, dose and total quantity) of the drug on the label? | 20 | 22.2 | 57.8 |
| Does the dispenser write frequency of administration on the label? | 44.4 | 2.2 | 53.3 |
| Does the dispenser write duration therapy of the drug on the label? | 42.2 | 2.2 | 55.6 |
| Does the dispenser write directions for use on the label? | 11.1 | - | 88.9 |
| Does the dispenser write date of dispensing on the label? | - | - | 100 |
| Does the dispenser write storage condition of drugs on the label? | - | - | 100 |
